# Supplementary material for: Cellular and Molecular Immunity to Influenza Viruses and Vaccines
Source: Vaccines (Basel). 2024 Apr 7;12(4):389. doi: 10.3390/vaccines12040389 (PMC11154265; doi:10.3390/vaccines12040389)
Supplement: Supplementary file 1 [file vaccines-12-00389-s001.zip › vaccines-2946665-supplementary.pdf]

Figure S1

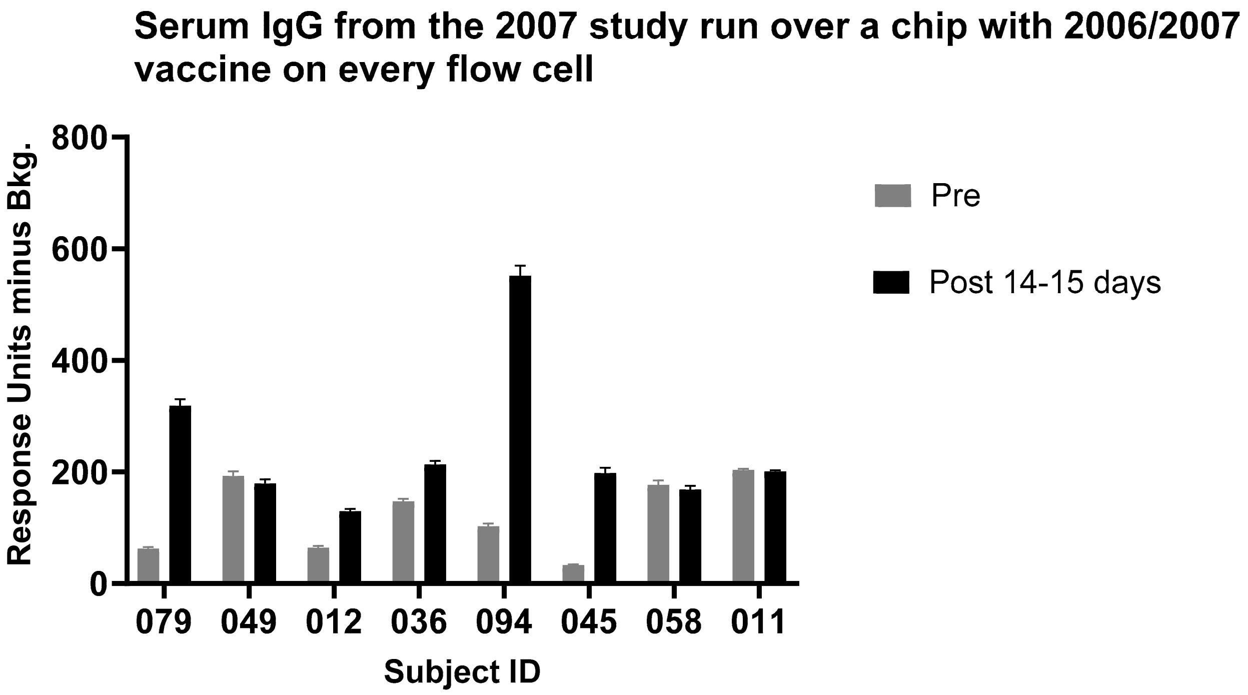

The vaccine from the 2006/2007 season was attached by amine coupling to a CM5 chip at ~5000 units on flow cells, F2, F3, and F4. Flow cell F1 was used as a background binding negative control. Purified IgG (both pre- and post-14-15 days) at 500 µg/ml from each subject was injected over the chip as described in the methods section. Bar graphs represent average RU minus background RU ± Standard Error (SE) for each subject.

Figure S2

A

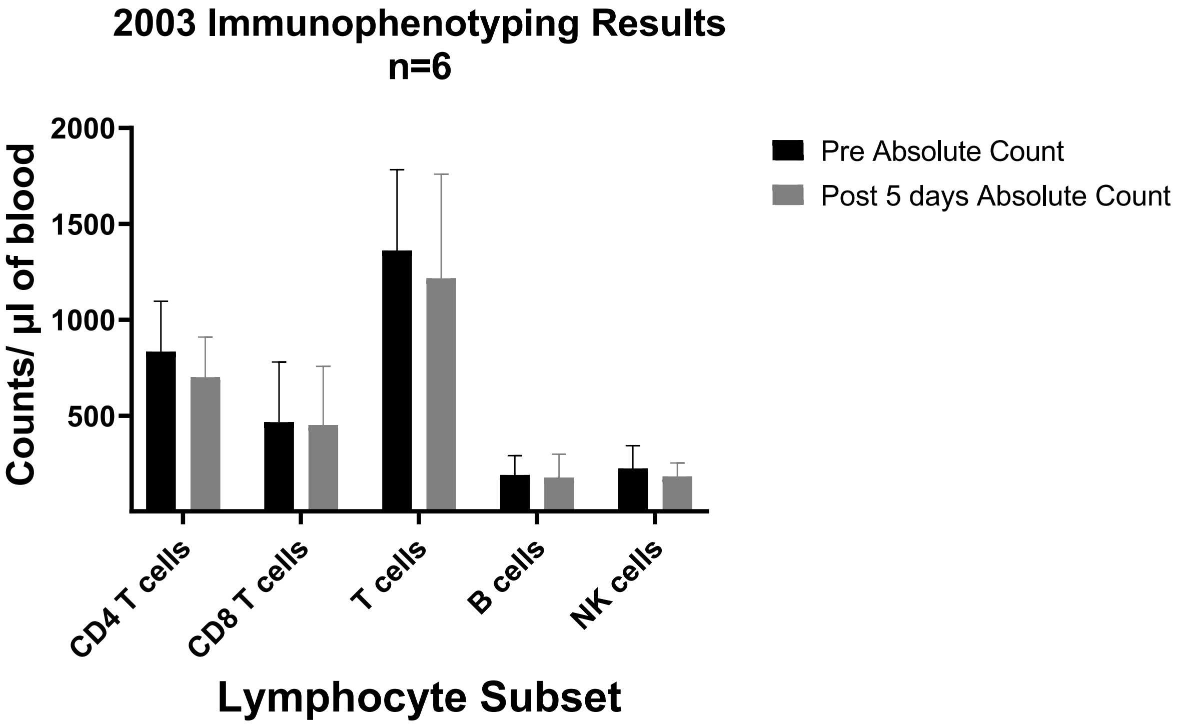

2003 Immunophenotyping Results
n=6
2000
1500
1000
500
Counts/ µl of blood
CD4 T cells
CD8 T cells
T cells
B cells
NK cells
Lymphocyte Subset
Pre Absolute Count
Post 5 days Absolute Count

B

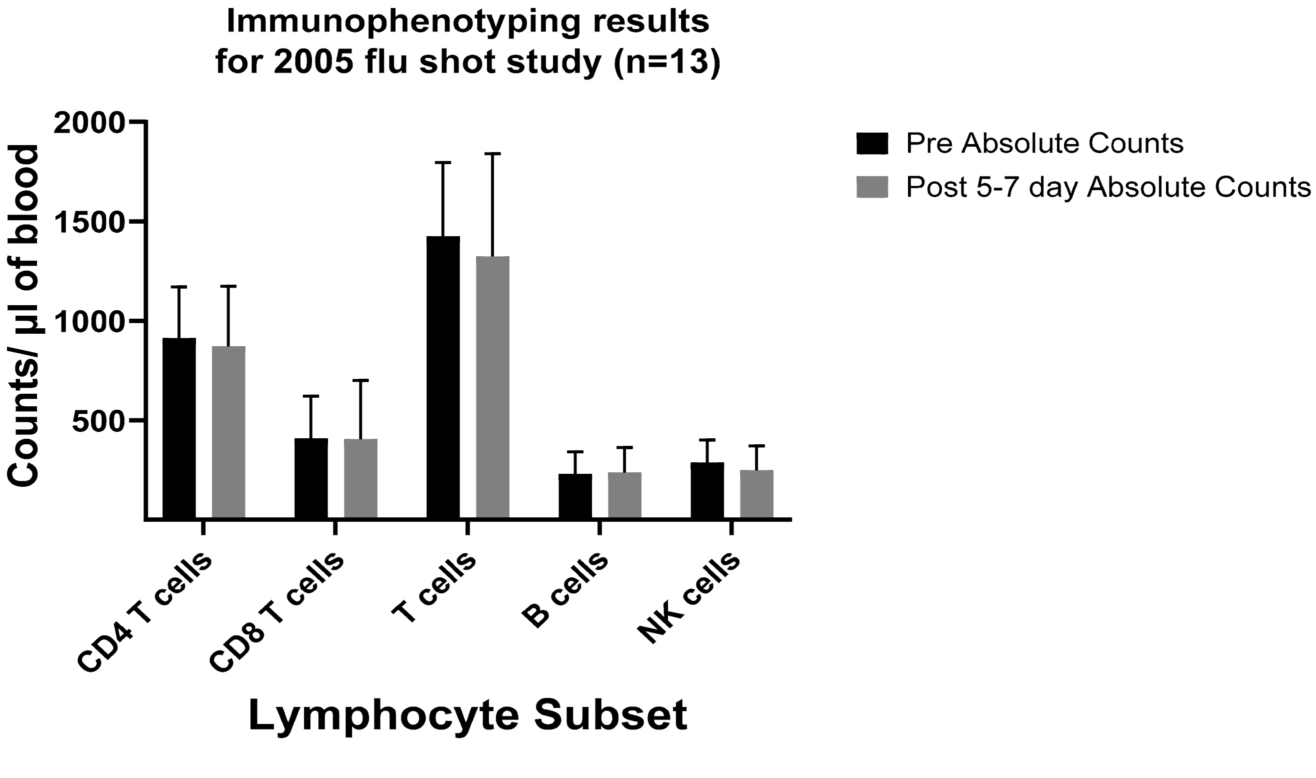

Immunophenotyping results
for 2005 flu shot study (n=13)
2000
1500
1000
500
Counts/ µl of blood
CD4 T cells
CD8 T cells
T cells
B cells
NK cells
Lymphocyte Subset
Pre Absolute Counts
Post 5-7 day Absolute Counts

Immunophenotyping results from the 2003/2004 (A) and 2005/2006 (B) flu seasons. Study subjects were immunophenotyped with the 4-color MultiTest reagents with Trucount tubes from BD Biosciences according to the manufacturer's protocol. Stained samples were run and analyzed on a FACSCalibur flow cytometer with MultiSet software v1.1.1 (BD Biosciences, San Jose, CA, USA). Graph bars indicate average counts ± SE.

Figure S3: Gating Strategy for $CD4^+$ and $CD8^+$ T cells, Naive and Memory Cells

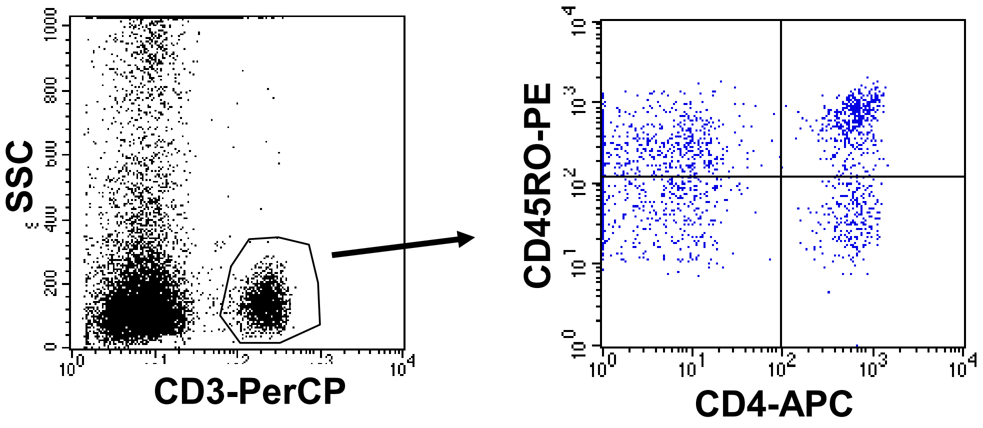

The method for determining the percentage of memory $CD4^+$ and $CD8^+$ T cells. In a plot of CD3 versus side scatter the $CD3^+$ cells are gated upon, and this gate is applied to a plot of $CD4^+$ versus $CD45RO^+$ (memory) cells. In this plot the quadrant statistics give the percentages of memory and naïve cells for $CD4^+$ and $CD8^+$ T cells. Analysis by CellQuest software v3.3 (BD Biosciences, San Jose, CA, USA).

Figure S4: Gating Strategy for HLA-DR+/CD38+/CD4+ T cells

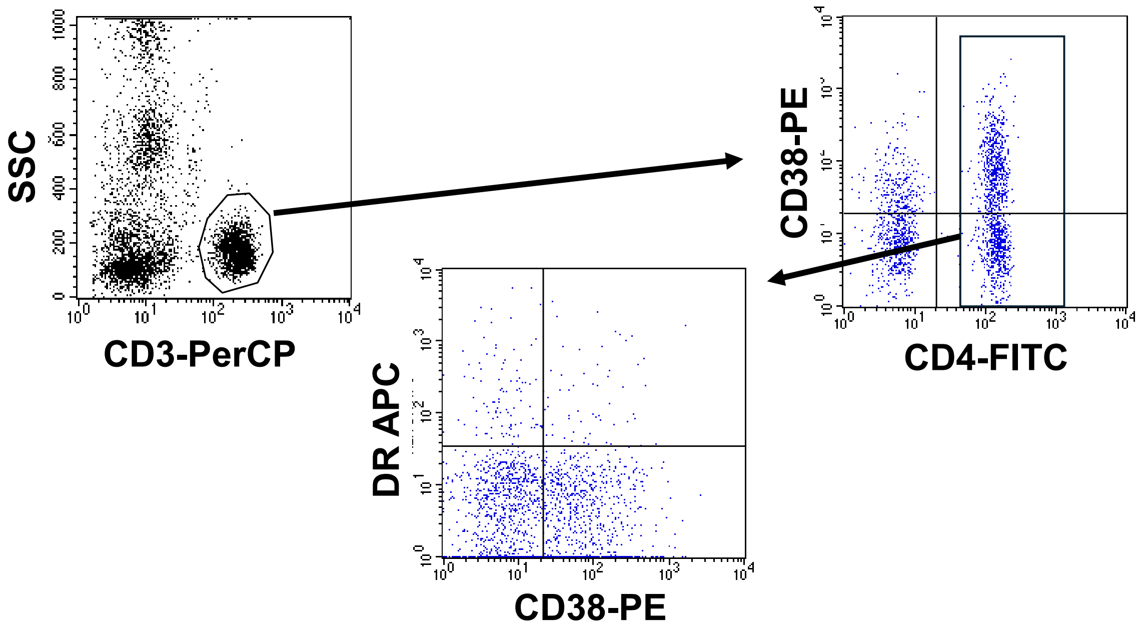

The method for determining the percentage of CD38+ and HLA-DR+ CD4+ T cells. In a plot of CD3+ versus side scatter the CD3+ cells are gated upon, and this gate is applied to a plot of CD4+ versus CD38+ cells. In this plot the CD4+ T cells are gated upon, and this gate is applied to a plot of CD38+ versus HLA-DR+ cells. Quadrant statistics will give the percentage of CD38+/HLA-DR+/CD4+ T cells. Analysis by CellQuest software v3.3.

Figure S5: Gating Strategy for CD4$^+$ T cell Naïve, Effector Memory, and Central Memory Cells

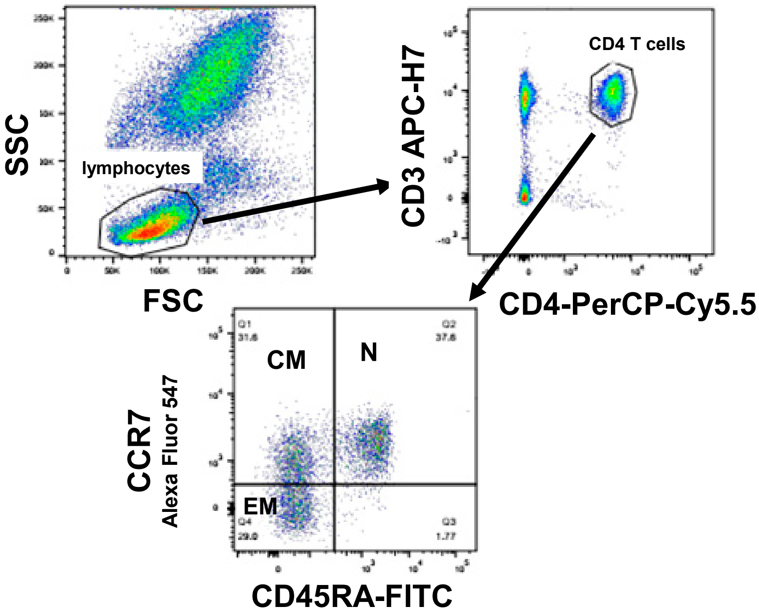

The method for determining the percentage of naïve (N), effector memory (EM), and central memory (CM) CD4$^+$ T cells. In a plot of forward scatter versus side scatter the lymphocytes are gated upon and this gate was applied to a plot of CD4$^+$ versus CD3$^+$ cells. In this plot the CD4$^+$ T cells were gated. This gate was applied to a plot of CD45RA versus CCR7 to identify naïve (N), effector memory (EM) and central memory cells (CM). The quadrant statistics gave the percentages of each CD4$^+$ T cell subset. Analysis by FlowJo software v10.7.1_CL (Tree Star. Inc. Oregon, USA).
